# Validation of commercial business lists as a proxy for licensed alcohol outlets

**DOI:** 10.1186/s12889-017-4419-0

**Published:** 2017-05-19

**Authors:** Heather A. Carlos, Joy Gabrielli, James D. Sargent

**Affiliations:** 1 Norris Cotton Cancer Center, C. Everett Koop Institute, Dartmouth College, Lebanon, NH 03756 USA; 2 Pediatrics, Geisel School of Medicine at Dartmouth, Lebanon, NH 03756 USA; 3 Department of Biomedical Data Science, Geisel School of Medicine at Dartmouth, Lebanon, NH 03756 USA; 4 Norris Cotton Cancer Center, One Medical Center Drive, Lebanon, NH 03756 USA

**Keywords:** Alcohol outlet, Commercial business list validity

## Abstract

**Background:**

Studies of retail alcohol outlets are restricted to regions due to lack of U.S. national data. Commercial business lists (BL) offer a possible solution, but no data exists to determine if BLs could serve as an adequate proxy for license data. This paper compares geospatial measures of alcohol outlets derived from a commercial BL with license data for a large US state.

**Methods:**

We validated BL data as a measure of off-premise alcohol outlet density and proximity compared to license data for 5528 randomly selected California residential addresses. We calculated three proximity measures (Euclidean distance, road network travel time and distance) and two density measures (kernel density estimation and the count within a 2-mile radius) for each dataset. The data was acquired in 2015 and processed and analyzed in 2015 and 2016.

**Results:**

Correlations and reliabilities between density (correlation 0.98; Cronbach’s α 0.97–0.99) and proximity (correlations 0.77–0.86; α 0.87–0.92) measures were high. For proximity, BL data matched license in 55–57% of addresses, overstated distance in 19%, and understated in 24–26%.

**Conclusions:**

BL data can serve as a reliable proxy for licensed alcohol outlets, thus extending the work that can be performed in studies on associations between retail alcohol outlets and drinking outcomes.

## Background

Retail alcohol outlets determine availability of alcohol for purchase, and availability is related to alcohol consumption. From a policy standpoint, retail alcohol outlets are of interest because community and state regulations may dictate aspects of the sale of alcohol [1, 2]. Limits on outlets could curtail excessive alcohol consumption by increasing cost and limiting opportunities for social aggregation, physical access, and exposure to alcohol marketing. One research summary found greater density of alcohol outlets was associated with increased alcohol consumption and related harms, and subsequently, The Task Force on Community Preventive Services “found sufficient evidence of a positive association between alcohol outlet density and excessive alcohol consumption and related harms to recommend limiting alcohol outlet density through the use of regulatory authority (e.g., licensing and zoning) as a means of reducing or controlling excessive alcohol consumption and related harms” [3, 4].

The notion that limits on retail alcohol density could affect drinking is not without controversy, as findings from density studies have varied. A more recent systematic review [5] applied a quality assessment tool to 26 publications that investigated associations between community availability of alcohol and alcohol use. Methodological heterogeneity precluded a meta-analysis; thirteen studies on outlet density and two on distance to nearest outlet included a range of exposure measures. Results from studies on the influence of availability of alcohol from commercial sources on drinking were also heterogeneous. For alcohol outlet density, authors found better evidence for associations with density of on-premise compared to off-premise outlets, and sparse evidence that proximity to alcohol outlets was important. Many studies had many participants in each cluster, with few or no individual-level covariates, raising concerns about the ecological fallacy [6]. Authors of this review deemed the literature inconclusive.

Publications included in both reviews were conducted primarily in North America. Only 2 of the US publications [7, 8] were national in scope, and were limited to just 32 and 8 college campuses, respectively. Thus, regional differences in how retail alcohol outlets are regulated may also contribute to the heterogeneity of the findings.

The lack of U.S. national studies examining impacts of alcohol outlet density and proximity on alcohol consumption remains a clear gap in the literature. A primary research barrier is the lack of national data on the location of alcohol outlets, in part because alcohol outlets are regulated by state and local jurisdictions. Some areas make license data readily available, but no national database listing all alcohol outlets exists. Since alcohol sales are regulated, they tend to occur in similar types of establishments (liquor stores, grocery stores, etc.) and thus commercial business lists (BL) may be able to serve as a secondary data source for licensed outlets. To explore this possibility, we compared alcohol outlet proximity and density measures derived from BL and state license data for a random sample of California residential addresses.

## Methods

### Study site

We selected the state of California as our study site because of its large size, easy availability of licensed alcohol outlet data, and the diversity of both urban and rural areas.

### Alcohol outlets

Licensed alcohol outlets are classified as either on-premise or off-premise based on where the purchased alcohol is consumed. On-premise outlets are primarily restaurants and bars. Information from a BL is not sufficient to determine if a restaurant sells alcohol (even some Burger Kings sell beer), and, although bars are identified in a BL, there is not always clear distinction between bars and restaurants. Thus, we limited our study to off-premise alcohol outlets.

*Commercial business list* (BL) data was obtained from OneSource’s (now Avention) Global Business Browser during April 2015. Avention is a business information service which aggregates business lists from over 2500 data sources including primary research, regulatory filings, corporate web sites, company press statements, annual reports, news stories, and analyst research, and is considered one of the most comprehensive sources of national business list data. Probable alcohol retailers were identified using the North American Industry Classification System (NAICS) codes. NAICS is the standard used by Federal statistical agencies to classify businesses based on their primary activity [9]. Business names and addresses were downloaded for the following NAICS codes: 44511 supermarkets and other grocery stores, 44512 convenience stores, 44531 beer, wine, and liquor stores, 44611 pharmacies and drug stores, 44711 gasoline stations with convenience stores, 44719 other gasoline stations, 452111 department stores, 452112 discount department stores.

For data quality control, we first queried (via website or phone call) all pharmacies with more than 15 locations in California (48% of all pharmacies) and department stores with 10 or more locations (81% of all stores) to determine if they sold alcohol and included/excluded them accordingly. Based on this analysis, we excluded all other department stores and pharmacies as not likely to sell alcohol. While many discount department stores are small stores that do not sell alcohol, nationally, we have seen warehouse stores that do sell alcohol included in this NAICS category. Therefore, we scanned the names of discount department stores with 2 or more locations (13% of all stores) for large retailer names. The BL data contained duplicate records for some businesses, such as a pharmacy in a grocery store or slight discrepancies in business names. We compared geocoded locations, business names and phone numbers and removed locations that appeared to be duplicates.

We downloaded 39,186 potential alcohol outlets from the BL. We identified 7886 establishments not likely to sell alcohol and 1088 that were duplicate listings, which resulted in 30,212 alcohol outlets identified from the BL.

*Licensed* alcohol retailers were obtained from the California Department of Alcohol Beverage Control (data was refreshed on April 1, 2015). We limited our license data to off-premise retail outlets with fixed locations. These license types included: 20 Off-Sale Beer and Wine, 21 Off-Sale General, and 85 Limited Off-Sale Retail Wine License. We then reviewed the status of each license and removed all that were not active or in good standing (status of Surend (surrender), RevPen (review pending) Suspen (suspend), and Pend (pending)).

For licensed outlets, there were 31,607 outlets with off-premise licenses; however 4103 of these were not active or in good standing, which left a total of 27,504 licensed alcohol outlets.

### Residential addresses

We purchased 6000 randomly selected California residential addresses from AccuData Integrated Marketing, a company that sources their address data from the United States Postal Service. Since there are likely difference in the distribution of alcohol outlets in urban and rural areas, we classified each address as urban/rural starting with a 4-tier scheme [10] derived from the Rural-Urban Commuting Area (RUCA) classification system [11]. The RUCA system considers commuting patterns to nearby areas. A RUCA category (urban core, sub-urban, large rural town and small town/isolated rural) was assigned to each residential address based on their geocoded location. Given the paucity of residential addresses in the 2 rural categories (large rural town *n* = 77 and small town/isolated rural *n* = 36), we combined these into one “rural” category. To ensure that addresses were representative, we compared address counts by county and RUCA category to population distributions for all of California. Our sample of 6000 residential addresses covered 57 of California’s 58 counties and was reasonably proportionally representative of the population in these counties (e.g. Los Angeles County contains 26.2% of California’s population and our sample had 1526 or 25.4% of the 6000 addresses were located in that county).

### Geocoding

Retail outlet addresses from BL and license data as well as the residential addresses were geocoded using 2013 StreetMap N.A [12] using ArcGIS v.10.3.1 (ESRI, Redlands, CA). Outlet addresses were first geocoded to the street address, and, if no match was found, they were geocoded to the ZIP code centroid. Residential addresses were geocoded to the street address; if no match was found, they were considered unmatched.

Both BL and licensed alcohol outlets had similar rates of geocoding, with only 0.3% and 1.2% of the outlets not matched, respectively. Over 97% of license and 98% of BL addresses were geocoded to their street address. The remaining ~1% of each dataset was geocoded to the ZIP code centroid.

Just 459 (7.7%) of the 6000 residential addresses were not matched to the street address, 8 were duplicates (e.g. different apartments at the same address) and 5 had network measures (see below) that could not be calculated, giving a final count of 5528 residential addresses.

### Measures

We selected measures that are often used in studies involving individuals and proximity and density of outlets (alcohol, tobacco and food; e.g. [7, 13, 14]). We purposely excluded measures that used administrative boundaries (e.g. ZIP codes, Census tracts), as they impose constraints not observed by study subjects (people do not base daily travel on administrative boundaries and often lack awareness of where boundaries are). All measures were calculated using the geocoded Residential Address using ArcGIS v.10.3.1, and the same measures were calculated for both BL and license data.

#### Proximity measures

*Euclidean distance* is the straight line distance from the residential address to the nearest alcohol outlet measured in miles.

*Network* measures minimize distance traveled along a street network (in this case StreetMap N.A. [12]) to the closest alcohol outlet. Both distance in miles (Network Distance) and the estimated driving time in minutes (Network Time) are reported.

#### Density measures

*Kernel density estimation* [15] (KDE) fits a probability density function over each alcohol outlet such that the value is highest at the outlet and zero at a specified distance (5 miles in this case). Each pixel within the 5-mile radius is assigned a value based on this density function (outlets per square mile). The final value assigned to each pixel in the study area is the sum of the KDE values for each alcohol outlet within 5 miles of the pixel. Geocoded addresses are then overlain on the KDE raster, and the value of the KDE raster pixel that aligns with the residential address is the KDE value assigned to that address.

*2 Mile Radius* is a count of alcohol outlets within 2 miles (Euclidean distance) of the residential address.

### Statistical analysis

We calculated correlation coefficients and the inter-correlation (Cronbach’s α reliability coefficients; a measure of internal consistency) between license and BL data for each measure for the entire set of residential addresses and across the 3 RUCA categories. Based on distributional properties of our measures (significant positive skew) we provided Pearson’s correlation coefficients to address limitations in Cronbach’s α when used with non-normally distributed data. In addition, for the proximity measures, we performed a Wilcoxon Signed-Rank Test to assess how measures differed and the direction of mismatch. This approach provided further information about the number of exact matches as well as how frequently BL data rankings overestimated versus underestimated license data rankings. Statistical analysis was performed using STATA v.12.1 (StataCorp LP, College Station, Texas) and results were visualized in ArcGIS v. 10.3.1 (ESRI, Redlands, CA) as needed.

## Results

Descriptive statistics are presented in Table 1. Medians and first and third quartiles (Q1 and Q3) were used as summary statistics, as variables were positively skewed. Medians and first and third quartiles were similar between BL and license data, particularly for proximity measures. For density, medians for BL were 10–20% higher than for license data. The majority (91.5%) of addresses were classified as urban core, which mirrors the population of California (89.7% urban). As expected, proximity values increased (addresses were further from alcohol outlets) as rurality increased, whereas density values were greatest in the urban core and lowest in rural settings.

**Table 1:** Descriptive statistics

N (%)	All	Rural	Sub-Urban	Urban Core
	5528 (100%)	113 (2.0%)	356 (6.4%)	5059 (91.5%)
	Median [Q1,Q3]	Median [Q1,Q3]	Median [Q1,Q3]	Median [Q1,Q3]
Proximity				
BL Euclidean (miles)	0.28 [0.15,0.51]	0.70 [0.26,1.76]	0.53 [0.28,1.33]	0.27 [0.15,0.48]
License Euclidean (miles)	0.29 [0.16,0.51]	0.70 [0.26,1.75]	0.54 [0.27,1.28]	0.28 [0.15,0.48]
BL Network Time (minutes)	1.16 [0.62,2.09]	2.40 [1.05,6.46]	2.27 [1.14,4.95]	1.11 [0.59,1.94]
License Network Time (minutes)	1.17 [0.63,2.10]	2.51 [1.00,6.59]	2.30 [1.12,4.88]	1.12 [0.61,1.94]
BL Network Distance (miles)	0.45 [0.24,0.81]	1.01 [0.36,2.44]	0.83 [0.42,1.91]	0.43 [0.23,0.76]
License Network Distance (miles)	0.45 [0.24,0.82]	0.97 [0.35,2.40]	0.83 [0.40,1.81]	0.43 [0.24,0.76]
Density				
BL KDE (outlets per sqmi)	3.07 [1.48,5.85]	0.35 [0.09,0.70]	0.48 [0.16,0.90]	3.43 [1.78,6.19]
License KDE (outlets per sqmi)	2.60 [1.36,4.64]	0.33 [0.11,0.76]	0.47 [0.18,0.90]	2.90 [1.62,4.85]
BL 2 Mile Radius (count)	46 [21,84]	7 [1,16]	8 [1,18]	51 [26,88]
License 2 Mile Radius (count)	40 [20,67]	6 [1,15]	9 [2,19]	44 [23,70]

Density and proximity measures for BL and license data were highly correlated (Table 2). Correlation coefficients ranged from 0.77–0.98 (Table 2), with the highest correlations found for the density measures. Correlations by RUCA category ranged from a low of 0.47 for rural network distance to a high of 0.98 in urban core KDE. Rural areas (*n* = 113) had the lowest correlation coefficients for proximity measures while sub-urban areas were slightly lower for density measures. Similar levels of reliability estimates were demonstrate across license and BL measures (Cronbach’s α = 0.97–0.99 for density measures and α = 0.87–0.92 for proximity measures). The range of coefficients reflects both the small sample size and variation in rural environments.

**Table 2:** Correlation coefficients with 95% confidence intervals and Cronbach’s Alpha reliability coefficients

	All	Rural	Sub-Urban	Urban Core
	(*N* = 5528)	(*n* = 113)	(*n* = 356)	(*n* = 5059)
Proximity	*r[95% CI]*; *α*	*r[95% CI]*; *α*	*r[95% CI]*; *α*	*r[95% CI]*; *α*
Euclidean Distance(miles)	0.83 [0.82, 0.84]; 0.90	0.75 [0.66, 0.82]; 0.85	0.82 [0.78, 0.85]; 0.89	0.82 [0.81, 0.83]; 0.90
Network Time (minutes)	0.86 [0.85, 0.86]; 0.92	0.66 [0.55, 0.76]; 0.90	0.84 [0.80, 0.87]; 0.90	0.90 [0.90, 0.91]; 0.95
Network Distance (miles)	0.77 [0.76, 0.78]; 0.87	0.47 [0.32, 0.60]; 0.91	0.84 [0.81, 0.87]; 0.91	0.85 [0.84, 0.86]; 0.92
Density				
KDE (outlets per sqmi)	0.98 [0.98, 0.98]; 0.97	0.91 [0.87, 0.94]; 0.94	0.89 [0.86, 0.91]; 0.94	0.98 [0.98, 0.98]; 0.96
2 mi Radius (count)	0.98 [0.97, 0.98]; 0.99	0.91 [0.88, 0.94]; 0.94	0.89 [0.87, 0.91]; 0.94	0.97 [0.97, 0.98]; 0.97

Wilcoxon Signed-Rank tests (Table 3) revealed that for proximity measures, BL data ranking provided an exact match for license data ranking 55–57% of the time. The BL data overestimated license measures 19% of the time and underestimated them 24–26% of the time. The actual values of overestimation and underestimation tended to be small, however, with median differences between datasets ranging from −0.10 to 0.13 miles (Euclidean and Network Distance) and −0.19 to 0.27 min for Network Time. As would be expected, median differences were smallest for urban core areas and slightly larger for sub-urban and rural addresses. In evaluation of ranking values, the distribution between BL tying and overestimating and underestimating the license measure was fairly consistent across RUCA categories.

**Table 3:** Wilcoxon signed-rank test. Values and median difference scores across BL and license data

		All (*N* = 5528)	Rural (*n* = 113)	Sub-Urban (*n* = 356)	Urban Core (*n* = 5059)
		*n* (%)	*n* (%)	*n* (%)	*n* (%)
		Median [Q1,Q3]	Median [Q1,Q3]	Median [Q1,Q3]	Median [Q1,Q3]
Euclidean Distance (miles)	Tied	3036 (55%)	64 (57%)	183 (51%)	2789 (55%)
	BL > License	1073 (19%)	30 (27%)	103 (29%)	940 (19%)
		0.09 [0.02, 0.23]	0.23 [0.06, 1.59]	0.16 [0.04, 0.80]	0.08 [0.02, 0.21]
	BL < License	1419 (26%)	19 (17%)	70 (20%)	1330 (26%)
		−0.06 [−0.17,-0.02]	−0.11 [−0.61,-0.04]	−0.24 [−0.66,-0.07]	−0.05 [−0.16,-0.02]
Network Time (minutes)	Tied	3134 (57%)	67 (59%)	196 (55%)	2871 (57%)
	BL > License	1039 (19%)	29 (26%)	93 (26%)	917 (18%)
		0.27 [0.09, 0.77]	0.70 [0.14, 2.72]	0.57 [0.19, 2.64]	0.25 [0.09, 0.68]
	BL < License	1355 (24%)	17 (15%)	67 (19%)	1271 (25%)
		−0.19 [−0.47,-0.06]	−0.32 [−2.90,-0.08]	−0.54 [−2.80,-0.20]	−0.18 [−0.44,-0.06]
Network Distance (miles)	Tied	3134 (57%)	67 (59%)	196 (55%)	2871 (57%)
	BL > License	1056 (19%)	29 (26%)	93 (26%)	934 (18%)
		0.13 [0.05, 0.34]	0.28 [0.08, 1.74]	0.28 [0.08, 1.19]	0.12 [0.04, 0.30]
	BL < License	1338 (24%)	17 (15%)	67 (19%)	1254 (25%)
		−0.10 [−0.23,-0.03]	−0.18 [−0.89,-0.05]	−0.25 [−1.26,-0.10]	−0.09 [−0.21,-0.03]

**Fig. 1:**
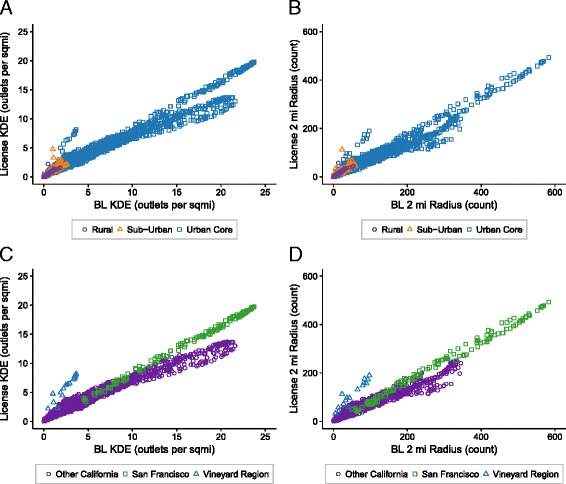
Scatterplots of license and BL density measures illustrating KDE (a and c) and 2 mi radius (b and d), by RUCA category (a and b) and by regions (c and d)

The scatterplots in Fig. 1 provide a visual depiction of the association between BL and license data for the two density measures. Strong positive linear associations reveal consistency across measurement approaches. Figure 1a (KDE) and Fig. 1b (2 mile radius) highlight the density measures by RUCA category, showing that urban areas provide all of the points for any KDE density beyond 5 outlets per square mile. Fig. 1a (KDE) and Fig. 1d (2 mile radius) reveal differences in the association between license and BL for San Francisco and Vineyard Regions, compared to the rest of California.

The scatter plots in Fig. 1c, d show three distinct patterns. The trajectories of linear fit for BL vs. license data points for San Francisco and the Vineyard Region are visually different from the rest of the state, with both regions having higher slopes (greater density of licensed outlets for each additional BL outlet) compared to the rest of California. San Francisco had the greatest density of both licensed and BL outlets despite the boundary effect [16]. San Francisco is on a peninsula and the alcohol outlets stop at the coast, yet the density calculations, which are based on 5 mile (KDE) and 2 mile (2 mile count) radii, do not consider this natural boundary.

The other geographic area that represented a potential outlier on the scatterplots in Fig. 1 was Napa Valley and surrounding areas in California’s wine country. Some vineyards have retail alcohol outlets located on site and these are not represented in the BL data. There is a NAICS code for vineyards, but there is no indication if the vineyard has a retail operation or, as in most cases, just represents the agricultural side of the business. Thus, in order to be more conservative, we did not include vineyards in the BL data. However, a search of the license data for “vineyard” in the business name showed 431 (out of 27,504 total licensed outlets), most of which were not represented in the BL data. The 15 residential addresses that are highlighted in Fig. 1c, d are addresses that have at least 20 vineyards within a 5 mile radius, and are all located in Napa County.

## Discussion

Because licensing data for alcohol outlets is not nationally available, we evaluated the utility and accuracy of commercial business list (BL) data as a proxy for licensed alcohol outlets. Correlations and inter-item reliability were strong for all measures, and values across BL and license data measures were largely similar. For density, BL returned results that were highly and linearly correlated but with values that were 10–20% higher compared to license data. That density is overstated should be considered when reporting density results, but high correlations between the two measures suggest that BL density can serve as a reliable proxy for licensed alcohol outlet density in correlational studies. With respect to proximity, BL returned the exact value for distance to the closest outlet more than half the time, and errors were equally distributed on either side of zero, suggesting that error was random. Thus, results from this study provide strong support for measurement equivalence and with particular strength for density measurement.

The scatterplots offer visual evidence of linear relations between BL and license outlet density. Slight differences in slope for data subsets may indicate possible regional variations in retail environments or boundary effects most notably in the urban core with some spill over to the sub-urban areas. The data categorized by RUCA reveal correlations and inter-item reliability estimates of the proximity and density measures were stronger in urban compared to rural areas. This is probably because rural areas have small, dispersed populations, so the licensed alcohol outlets were less numerous and errors of omission or substitution result in larger errors for density and proximity. Any national or large regional studies might consider stratifying analyses on urban/rural status, in part because there are such large differences in outlet density across these groups, and because estimates in rural areas seemed less reliable.

Our findings differ from studies of food food outlets [17–23] which have examined BL outlets by field verification and overwhelmingly found only modest levels of agreement. On the other hand, one study on tobacco outlets (in a non-licensing state) found the BL to be reasonably accurate [24]. Our approach accepts from the outset that BL data will overstate the total number of alcohol outlets (not every grocery store will sell alcohol) while also missing some actual alcohol outlets (e.g. a store that has a different NAICS code). Since licensed alcohol outlets in the vicinity of a residence are the outcome of interest, we did not compare individual BL outlets to individual licensed outlets as one would when ground-truthing (verifying the location/validity of the outlet by direct observation). Based on the high correlations demonstrated across BL and licensed data in the present analyses, findings from this study suggest that BL data is a strong proxy for licensed alcohol outlets, even without using the extremely time-consuming approach of ground-truthing.

## Limitations

Our study was focused on off-premise alcohol outlets. Although the license data did contain on-premise establishments (primarily bars and restaurants), it is difficult to discern these establishments in the BL data. There is a NAICS code: 722,410 Drinking Places (alcoholic beverages) that would likely identify most bars. Restaurants, recreational facilities (bowling, golf courses) and social organizations (e.g., Elks) that do and do not serve alcohol, however, are not grouped separately.

Overall and across rural, sub-urban and urban cores, density measures had stronger correlations than proximity measures. This was expected since density measures usually include a number of alcohol outlets whereas proximity measures are calculated based on the closest one. If the BL data had an additional “false” outlet or did not include a licensed outlet, this mismatch may be very apparent in determining the closest outlet, but not necessarily in a density measure which might include many outlets.

The number of rural addresses explored in this study was small (just 113 out of 5528), reflecting the small size of rural populations. Moreover, rural addresses cover a wide range of rural environments with respect to outlet density (the Q1 to Q3 range for 2 mi Radius was a count of 1–16 licensed alcohol outlets). Care should be taken when studying alcohol access in rural settings.

As discussed above, we identified two regions – San Francisco and Napa County where the alcohol outlet environment differed from the rest of California. All these differences were small, Care should be taken in applying our methods to other states or nationally to explore the data for unusual local retail environments that deviate from the norm. For example, many states have dry (no alcohol sales permitted) or moist (sales are restricted to certain areas) counties, and the BL data will need to be adjusted accordingly for those geographic areas.

## Conclusion

The purpose of this study was to evaluate use of BL data as a proxy for licensed alcohol outlets. This is of particular interest for areas where license data is not available or studies that cover large regions (several states or national) that may not have license data available. Our study in California suggests that BL data is a strong proxy for license data for alcohol outlets, especially in sub-urban and urban areas. This suggests that BL data could be used to study alcohol outlet geography and its correlation with drinking behaviors in national data sets. However, in order to use BL data in place of license data, care must be taken to understand and consider factors relevant for the retail alcohol environment throughout the study area.

## Abbreviations

BL: Commercial business list
KDE: Kernel density estimation
IQR: Inter-quartile range
Q1: First quartile
Q3: Third quartile
CI: Confidence interval
NAICS: North American Industry Classification System
RUCA: Rural urban commuting area

## References

[1] Paschall MJ, Grube JW, Black C, Ringwalt CL (2007). Is Commercial Alcohol Availability Related to Adolescent Alcohol Sources and Alcohol Use? Findings from a Multi-Level Study. J Adolesc Health.

[2] Stockwell T, Gruenewald PJ. Controls on the physical availability of alcohol. In: Heather N, Peters TJ, Stockwell T, editors. International Handbook of Alcohol Dependence and Problems Part VI Prevention of Alcohol Problems. Chichester, John Wiley & Sons; 2001.

[3] Task Force on Community Preventive S (2009). Recommendations for reducing excessive alcohol consumption and alcohol-related harms by limiting alcohol outlet density. Am J Prev Med.

[4] Campbell CA, Hahn RA, Elder R, Brewer R, Chattopadhyay S, Fielding J, Naimi TS, Toomey T, Lawrence B, Middleton JC (2009). The effectiveness of limiting alcohol outlet density as a means of reducing excessive alcohol consumption and alcohol-related harms. Am J Prev Med.

[5] Bryden A, Roberts B, McKee M, Petticrew M (2012). A systematic review of the influence on alcohol use of community level availability and marketing of alcohol. Health Place.

[6] Piantadosi S, Byar DP, Green SB (1988). The ecological fallacy. Am J Epidemiol.

[7] Scribner R, Mason K, Theall K, Simonsen N, Schneider SK, Towvim LG, DeJong W (2008). The Contextual Role of Alcohol Outlet Density in College Drinking. J Stud Alcohol Drugs.

[8] Weitzman ER, Folkman A, Lemieux K, MPH F, Wechsler H (2003). The relationship of alcohol outlet density to heavy and frequent drinking and drinking-related problems among college students at eight universities. Health & Place.

[9] Introduction to NAICS [https://www.census.gov/eos/www/naics/index.html ]. Accessed 21 Apr 2015.

[10] Guidelines for Using Rural-Urban Classification Systems for Public Health Assessment [http://www.doh.wa.gov/Portals/1/Documents/1500/RUCAGuide.pdf]. Accessed 17 May 2017.

[11] Rural-Urban Commuting Area Codes [http://www.ers.usda.gov/data-products/rural-urban-commuting-area-codes.aspx]. Accessed 2 Mar 2016.

[12] U.S. and Canada Detailed Streets. In: 2013 - StreetMap^™^ North America. Edited by TomTom North America, Inc. Redlands, California. USA: ESRI; 2013.

[13] Pollack CE, Cubbin C, Ahn D, Winkleby M (2005). Neighbourhood deprivation and alcohol consumption: does the availability of alcohol play a role?. Int J Epidemiol.

[14] Adachi-Mejia AM, Carlos HA, Berke EM, Tanski SE, Sargent JD: A comparison of individual versus community influences on youth smoking behaviours: a cross-sectional observational study. BBMJ Open 2012;2:e000767. doi: 10.1136/bmjopen-2011-000767. 10.1136/bmjopen-2011-000767 PMC3437428 22942229

[15] Carlos HA, Shi X, Sargent J, Tanski S, Berke EM (2010). Density estimation and adaptive bandwidths: a primer for public health practitioners. Int J Health Geogr.

[16] Lawson AB, Biggeri A, Dreassi E, Andrew L, Annabele B, Dankmar B, Emmanuel L, Jean-François V, Roberto B (1999). Edge Effects in Disease Mapping. Disease Mapping and Risk Assessment for Public Health.

[17] Caspi CE, Friebur R (2016). Modified ground-truthing: an accurate and cost-effective food environment validation method for town and rural areas. Int J Behav Nutr Phys Act.

[18] Fleischhacker SE, Rodriguez DA, Evenson KR, Henley A, Gizlice Z, Soto D, Ramachandran G (2012). Evidence for validity of five secondary data sources for enumerating retail food outlets in seven American Indian communities in North Carolina. Int J Behav Nutr Phys Act.

[19] Gustafson AA, Lewis S, Wilson C, Jilcott-Pitts S (2012). Validation of food store environment secondary data source and the role of neighborhood deprivation in Appalachia, Kentucky. BMC Public Health.

[20] Han E, Powell LM, Zenk SN, Rimkus L, Ohri-Vachaspati P, Chaloupka FJ (2012). Classification bias in commercial business lists for retail food stores in the U.S.. Int J Behav Nutr Phys Act.

[21] Liese AD, Colabianchi N, Lamichhane AP, Barnes TL, Hibbert JD, Porter DE, Nichols MD, Lawson AB (2010). Validation of 3 Food Outlet Databases: Completeness and Geospatial Accuracy in Rural and Urban Food Environments. Am J Epidemiol.

[22] Powell LM, Han E, Zenk SN, Khan T, Quinn CM, Gibbs KP, Pugach O, Barker DC, Resnick EA, Myllyluoma J (2011). Field validation of secondary commercial data sources on the retail food outlet environment in the U.S. Health Place.

[23] Seliske L, Pickett W, Bates R, Janssen I (2012). Field validation of food service listings: a comparison of commercial and online geographic information system databases. Int J Environ Res Public Health.

[24] D'Angelo H, Fleischhacker S, Rose SW, Ribisl KM (2014). Field validation of secondary data sources for enumerating retail tobacco outlets in a state without tobacco outlet licensing. Health Place.

